# Understanding TR Binding to pMHC Complexes: How Does a TR Scan Many pMHC Complexes yet Preferentially Bind to One

**DOI:** 10.1371/journal.pone.0017194

**Published:** 2011-02-22

**Authors:** Javed Mohammed Khan, Shoba Ranganathan

**Affiliations:** 1 Department of Chemistry and Biomolecular Sciences and ARC Centre of Excellence in Bioinformatics, Macquarie University, Sydney, Australia; 2 Department of Biochemistry, Yong Loo Lin School of Medicine, National University of Singapore, Singapore, Singapore; Dana-Farber Cancer Institute, United States of America

## Abstract

Understanding the basis of the binding of a T cell receptor (TR) to the peptide-MHC (pMHC) complex is essential due to the vital role it plays in adaptive immune response. We describe the use of computed binding (free) energy (BE), TR paratope, pMHC epitope, molecular surface electrostatic potential (MSEP) and calculated TR docking angle (θ) to analyse 61 TR/pMHC crystallographic structures to comprehend TR/pMHC interaction. In doing so, we have successfully demonstrated a novel/rational approach for θ calculation, obtained a linear correlation between BE and θ without any “codon” or amino acid preference, provided an explanation for TR ability to scan many pMHC ligands yet specifically bind one, proposed a mechanism for pMHC recognition by TR leading to T cell activation and illustrated the importance of the peptide in determining TR specificity, challenging the “germline bias” theory.

## Introduction

For maximal immunological protection against a multitude of pathogens, the adaptive immune response in higher jawed vertebrates causes major histocompatibility complexes (MHC) or human leukocyte antigens (HLA) in human, to bind antigenic peptides (p) and present them as peptide-MHC (pMHC) complexes on the surface of antigen-presenting cells (APC), for recognition by T cell receptors (TR) [1]. This TR/pMHC interaction is relatively feeble compared to other important interactions between the molecules of the immune system [2], yet strong enough to trigger TR mediated activation of T cells, thereby eliciting an immediate immune response to either destroy infected cells directly (*via* CD8+ associated cytotoxic T cells) or activate (*via* CD4+ associated helper T cells) other immune system cells like B cells and macrophages to carry out the immune response. More than ten years after the first TR/pMHC structure was reported [3], the interaction between TR and pMHC complexes is still an enigma [4], due in part to the complexities of the molecules involved in this association. The two constant domains (Cα and Cβ) of the TR are linked to variable domains (Vα and Vβ encoded by rearranged variable (V), diversity (D) and joining (J) genes, V-J and V-D-J genes, respectively), whose CDR1, CDR2 and CDR3 loops recognize pMHC [5]. The MHC proteins are composed of two chains, α and β, with the α chain (I-ALPHA) alone forming the peptide-binding groove in MHC class I (MHC-I) proteins, while MHC class II (MHC-II) proteins have both chains α (II-APLHA) and β (II-BETA) forming the peptide binding site [6].

The mechanism responsible for the specificity of the TR/pMHC interactions remains an unsolved problem. The TR "germline bias", in which TR/pMHC binding is independent of the nature of the peptide and MHC restriction or TR specificity is based on specific conserved contacts between TR V (variable) domains and MHC proteins that co-evolve [7], has been proposed as one of the solutions. It however, is not as simple as it sounds. This is due to the mechanisms of combinatorial diversity and N-diversity of the variable domains of TR that create 1012 TR per individual [5], the very high number of MHC alleles and most of all a large number of antigenic peptides. The cross-reactivity of MHC proteins means that the TR briefly scans through several pMHC complexes before actually interacting with a specific one. While this brief scanning by the TR may provide an explanation for the feeble TR/pMHC interactions alluded to earlier, it becomes increasingly important to understand the minute aspects of this vital binding over a broad spectrum of data. Garcia and co-workers [4] have provided highly influential hypotheses using a dataset of 20 TR/pMHC structures, implying that the contacts between CDR1 and CDR2 loops of TR variable domains and MHC helices are germline-encoded leading to the conclusion that TR/pMHC binding is peptide independent. Also inferred in their study is that whatever the TR docking angle, the bound complexes have equivalent binding free energies (*ΔG*; referred to here as binding energy (BE) in kcal/mol) at “codon” or amino acid positions A, B and C (as depicted inset of Figure 2b in [4]). Therefore, the main questions we address in this work are: (1) whether there are specific energetically equivalent binding energy “codon” or amino acid positions associated with TR binding angles as suggested by Garcia *et al*., [4] and; (2) if the “germline bias” theory really holds good across a large dataset. While addressing these questions, we have also arrived at a possible answer to another lingering question in immunology, *viz.* how can a TR scan through many pMHC complexes and yet specifically bind to one?

We have analyzed the currently available non-redundant dataset of 61 TR/pMHC X-ray crystal structures from MPID-T2 database (http://biolinfo.org/mpid-t2) [8], which were originally obtained from the Protein Data Bank (PDB) [9] and verified with IMGT/3Dstructure-DB (http://www.imgt.org/3Dstructure-DB/), the reference database for immunoglobulins, T cell receptors and MHC structures [10], [11], to determine three major factors that greatly contribute to or influence TR/pMHC binding: (1) binding energy (BE) between TR and pMHC complexes [12]-[14]; (2) molecular surface electrostatic potential (MSEP) at TR and pMHC interfaces [15], [16] and; (3) angle formed by the major axis of TR and the linear axis of the cognate peptide when TR is bound to pMHC (TR docking angle in degrees; herein referred to as ‘θ’ when calculated and as ‘diagonal’ when obtained from literature) [4], [17]. Using *in vitro* immuno-assays, researchers have previously reported that weak BE between TR and pMHC complexes ascribe weak agonistic (T cell activating) properties to the pMHC complexes and *vice versa* [18]–[20]. This inference is based on the underlying idea that the strength of TR binding to pMHC plays a vital role in stabilizing the half-life of the TR/pMHC complex, consequently resulting in T cell signalling or activation. This significant finding laid the foundation for us to use BE as a useful parameter in discriminating weak-, moderate- and strong pMHC agonists. MSEP has been used in structure based drug design and in understanding protein-protein interactions by crystallographers for many years [21]. It has also been applied as a successful molecular descriptor for large assemblies of molecules such as microtubules and ribosome [22]. Not only does it include all major aspects of protein-protein interaction, it is also distinctive of molecular shapes. Therefore, we have employed MSEP as an analytical tool to dissect TR/pMHC interactions.

Using computed MSEP of pMHC and TR interacting interfaces we are able to successfully explain the common docking geometry of almost all TR proteins on their respective pMHC binding interfaces. We then discuss a linear correlation between calculated BE and θ, which provides an answer to our first question. A TR paratope (residues on TR interface that contact the pMHC) and pMHC epitope (residues on pMHC interface that contact the TR) analysis, with a focus on conserved residues among pMHC and TR interacting sequence patterns, was conducted in hope of finding certain broadly conserved structural determinants that would constitute the “smoking gun” of “MHC bias” [4]. Finally, we also discuss a new and valuable grouping (clustering) system for TR proteins based on their binding site similarities (from TR paratope analysis), pMHC recognition similarities (from pMHC epitope analysis) and similarities in MSEP displayed by their respective interacting pMHC interfaces (see Methods section for details). The results of MSEP similarity calculation at the pMHC interface along with our TR paratope and pMHC epitope analyses also suggest a weakening of “germline bias” theory over a larger dataset and highlight the significant role played by the peptide in determining TR specificity, thereby, providing an explanation to our second query. Our detailed results are as follows.

## Results

### BE as a determinant of weak-, moderate- and strong pMHC agonists

**Figure 1:**
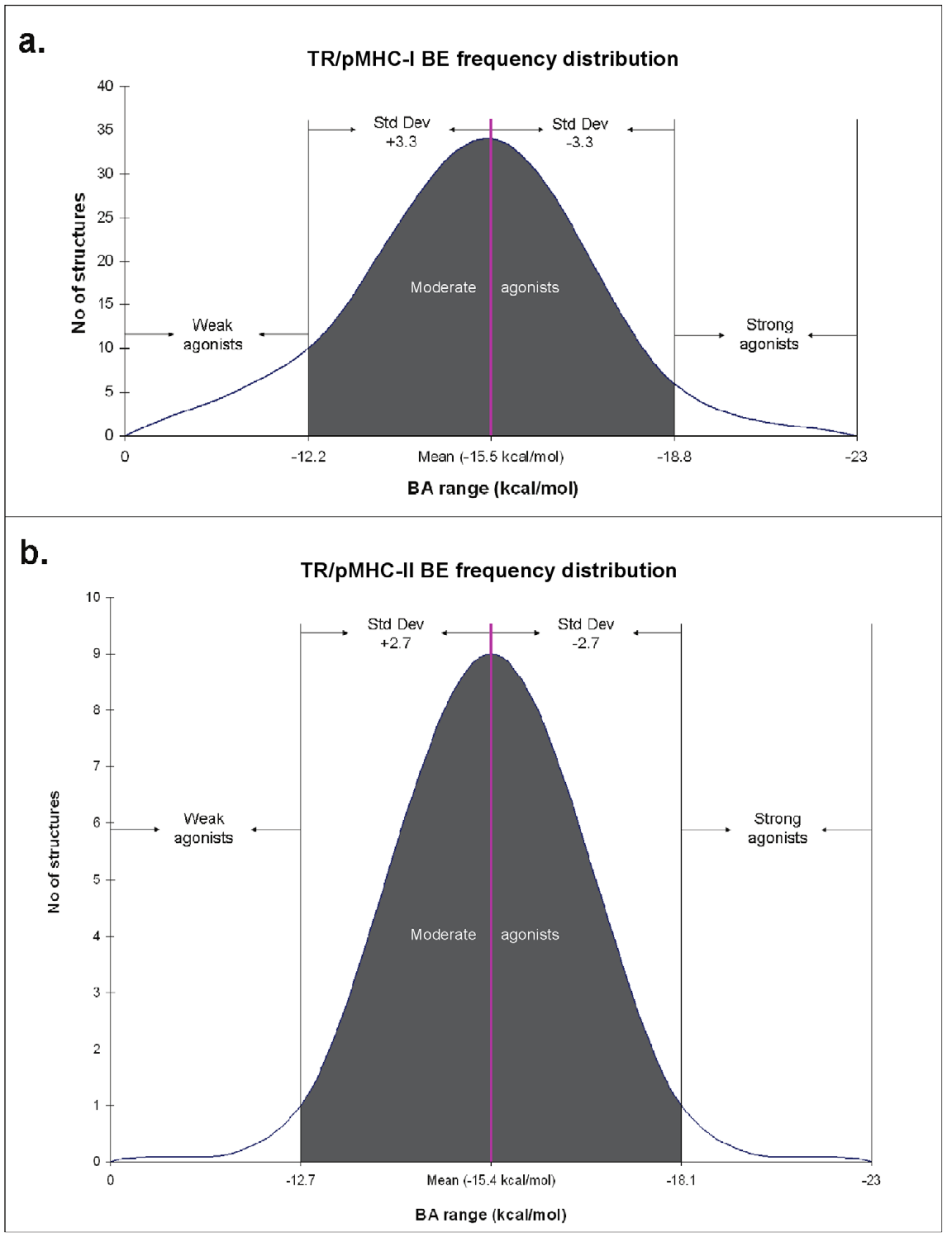
Standard curves for the frequency of computed BE between the TR and pMHC complexes for a. TR/pMHC-I complexes and b. TR/pMHC-II complexes. On the X-axis is the range of BE and on the Y-axis is the number of structures having their BE within these ranges. The pink lines signify the mean BE values. Standard deviation on either side of mean values is represented by shaded area (moderate agonists) in the graphs.

It has been reported earlier that lack of enough number of TR/pMHC structures makes differentiation of weak- and moderate-agonists from strong-agonists or true-agonists from antagonists, almost impossible without immunological assays [15]. However, the availability of a relatively large dataset (61 TR/pMHC structures) together with our comprehensive BE analysis has now made it possible to discriminate strong- from weak- and moderate-agonists for both TR/pMHC-I and TR/pMHC-II structures. Figure 1 shows a plot of the calculated BE between the TR and pMHC-I structures (Figure 1a) and pMHC-II structures (Figure 1b). As seen, this graphical representation gives a clear understanding of the discriminatory power of this analysis. We have computed an overall mean of -15.5 kcal/mol and −15.4 kcal/mol and standard deviation of ±3.3 kcal/mol and ±2.7 kcal/mol for TR/pMHC-I and TR/pMHC-II structures, respectively. With cutoffs defined by mean and standard deviation values, we have discriminated weak-, moderate- and strong pMHC agonists. Since BE is also referred to as binding free energy, the highest negative value is considered the best. Among TR/pMHC-I complexes, weak TR agonists have a BE between 0 and −12.2 kcal/mol ( = −15.5+3.3), moderate-agonists (shaded area in Figure 1a) have BE values between −12.2 and −18.8 kcal/mol ( = −15.5–3.3) while strong-agonists gave BE values below −18.8 kcal/mol and are potential T cell activators. TR/pMHC-II structures with a BE between 0 and −12.7 kcal/mol ( = −15.4+2.7) are classified as weak-agonists, complexes with BE between −12.7 and −18.1 kcal/mol ( = −15.4–2.7) are moderate-agonists (shaded area in Figure 1b) and strong-agonists have a BE value below −18.1 kcal/mol and could be more efficient in activating the T cells.

Figure 1a shows a few TR/pMHC-I complexes (PDB codes 1lp9, 2uwe, 2j8u, 2jcc, 3kpr and 3kps in Table S1) having BE values well below −20 kcal/mol, reaching up to −23 kcal/mol. These pMHC ligands are thus very strong-agonists with greater propensity to elucidate T cell activity, concordant with the results obtained from experimental immuno-assays by Miller *et al*. [23], for the pMHC ligands in the PDB structures 2uwe and 2jcc and Macdonald *et al*. [24], for the pMHC ligands in the PDB structures 3kpr and 3kps, respectively. Overall, it was observed that there were 10 (20%) weak-, 34 (68%) moderate- and 6 (12%) strong-binding agonists amongst the TR/pMHC-I complexes. The list of 34 moderate agonists includes pMHC ligands from the PDB structures 2ak4, 2bnr and 2nx5 (Table S1) which have been previously confirmed by cytotoxicity assays [25]–[27]. Among the 10 weak-agonists is the pMHC from the PDB structure 2ol3, whose lower propensity to elucidate T cell activity was validated by the low level of cytotoxicity observed from cytotoxicity assays by Mazza *et al*. [28]. Similarly, Figure 1b highlights the presence of one such strong-agonist (PDB code 3mbe in Table S1) amongst TR/pMHC-II structures with a BE of −22 kcal/mol. Observations made by Yoshida *et al*. [29], from functional immuno-assays clearly indicate the strong-agonistic and T cell stimulating properties of the pMHC complex in the PDB structure 3mbe. Amidst the 11 TR/pMHC-II complexes, our analysis established 1 (∼9%) weak-, 9 (∼82%) moderate- and 1 (∼9%) strong-binding agonist. These results suggest why a very small percentage (9–12% from our results) of peptide antigens that are predicted to be T cell epitopes by computational methodologies can actually elicit T cell response *in vitro* [30].

### pMHC interfaces display a ring of charged amino acids for recognition by complementarily charged TR Vα and Vβ domain interfaces

**Figure 2:**
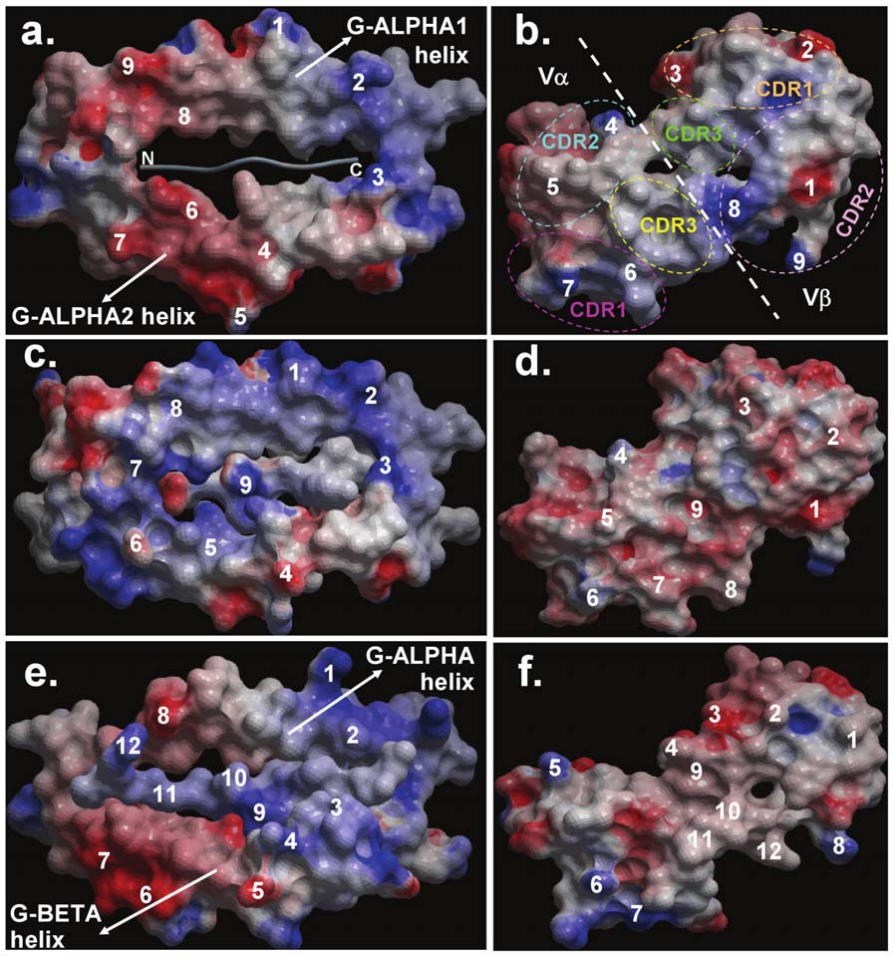
An aerial view of the MSEP displayed by the pMHC interfaces of TR/pMHC-I complexes a. 2e7l (PDB code), c. 1mwa (PDB code) and that of TR/pMHC-II complex e. 1u3h (PDB code) along with b, d, f. their respective contacting TR Vα and Vβ domain interfaces rotated 180° along their interacting axis to visualize their binding interface. The charged residues on the pMHC interfaces are numbered, which interact with the corresponding complementary charges (numbered accordingly) on their respective TR Vα and Vβ domain interfaces. These Vα and Vβ domain interfaces are collectively formed by the CDR1, CDR2 and CDR3 (shown as coloured dotted ovals in b.) loops that interact with the pMHC. The locations of CDR1, 2 and 3 loops in b. are the same for the TR interacting regions in **d**. and **f**.

Most TR proteins that recognize pMHC complexes bind on the central regions of G-ALPHA1 and G-ALPHA2 helices (Figure 2a) for pMHC-I and G-ALPHA and G-BETA helices (Figure 2e) for pMHC-II proteins [6]. MSEP displayed by the helices of a pMHC-I (PDB code 2e7l; Figure 2a) and pMHC-II (PDB code 1u3h; Figure 2e) clearly depict a sequential clockwise ring of positively and negatively charged residues on G-ALPHA1 and G-ALPHA2 helices (MHC-I), G-ALPHA and G-BETA helices (MHC-II) which interact with complementarily charged residues on CDR1 and CDR2 loops of TR α and β variable domains (Figure 2b, f). This was the case in almost all pMHC and TR interacting regions that were analyzed. Interestingly, previous characterization studies on TR/pMHC complexes have revealed molecular interactions along similar regions on the TR and pMHC interfaces [31], [32], thereby, supporting our MSEP driven interactions theory. However, in very few pMHC-I cases, such as 1mwa (PDB code), the MHC helices exhibit a ring of mostly positive residues with one/two negative residues on either helix contributing towards TR docking (Figure 2c). In such complexes, the corresponding binding TR interface is almost completely negatively charged, with one/two positive residues on either variable domain (Figure 2d). Across the entire dataset, the positive and negative arrangement seems to be by far more preferred than a ring with predominantly a single charge. It was also observed that negative charges on the two helices of both MHC-I and MHC-II structures occur around the N-termini of bound peptides whereas positive charges are located around their C-termini (Figure 2a, c and e).

A *vice versa* arrangement of charges is seen on TR interacting regions (Figure 2b, d and f). A noteworthy observation is that, MSEP presented by almost all pMHC interfaces are overall similar, suggesting that the ability of a TR to scan through many pMHC interfaces is attributable to the common electrostatic rings displayed on pMHC interfaces. Interestingly, a few, possibly key positions on pMHC interfaces vary in the charges displayed across the entire dataset. This is significant in the context of TR/pMHC interaction because mutating specific charged interacting residues on pMHC interfaces is known to cause increase or decrease in experimentally determined TR/pMHC binding affinity due to increased or decreased electrostatic interactions between the TR and pMHC leading to an enhanced or reduced T cell response, respectively [29]. As concluded in many earlier studies [16, 20, 28 and 33], our results confirm the importance of peptide in TR/pMHC binding, opposing the notion that TR/pMHC interaction is independent of peptide [4], [34]. A proof of this is the fact that various peptides display different combinations of positive and negative residues (Figure 2c and e) which interact with corresponding complementarily charged residues on highly variable CDR3 loops of TR Vα and Vβ domains (Figure 2d and f). Thus, the most variable regions of TR (CDR3) are positioned in the center of binding interface where they contact the peptide, whereas the more conserved regions of TR (CDR1 and CDR2) and the tops of MHC helices engage in contacts that surround the central CDR3-peptide region like a “gasket” [4]. Therefore, MHC helices along with bound peptides, present a set of electrostatic charges that are recognised by specific TR domains.

However, these surfaces should also not be too highly charged or they would bind other counter-ions that may need to be removed and hence might compete with TR for interaction. To support our theory, some short-(salt bridges) to long range (>4 Å distance) electrostatic interactions have been found in TR/pMHC crystal structures. For example, between the D10 TR Vα residue Lys68 (IMGT unique numbering {referred to as IMGT} 82; [35]) and murine MHC-II (I-Ak) G-BETA residue Asp76 (IMGT 72) in the PDB structure 1d9k [36] or between the A6 (PDB code 1ao7; [3]), B7 (PDB code 1bd2; [37]) and 2C (PDB code 2ckb; [38]) TR Vα residue Lys68 (IMGT 82) and the murine/human MHC-I (H2-Kb/HLA-A2) G-ALPHA2 residue Glu166 (IMGT 76) [6], [39]. Amongst other examples, are the electrostatic interactions between Glu52 (IMGT 63) residue of Vβ CDR2 loop and Arg79 (IMGT 79) residue of HLA-B8 in TR/pMHC-I complex LC13/EBV/HLA-B8 (PDB code 1mi5; [40]) and the interactions between the human MHC-II (HLA-DR1 and HLA-DR4; PDB codes 1fyt and 1j8h, respectively) G-ALPHA residue Lys39 (IMGT 43) (in a loop projecting up and away from the floor of β-sheet that forms the base of MHC binding groove) and the Vβ residue Glu56 (IMGT 67) of HA1.7 TR [16], [41]. A recent molecular modeling study proved that a single point mutation (G95R; IMGT 107) in Vβ CDR3 loop of 2C TR increased its affinity to QL9/Ld pMHC by a factor of 1000. This, they suggest, is most likely due to direct electrostatic interaction of Arg95 side chain with an Asp8 (IMGT 8) residue in the QL9 peptide nonamer [42]. Thus, electrostatic effects can work at a distance [43], especially for orienting purposes, so their role in orienting TR relative to pMHC at an early stage during antigen recognition is vital.

**Figure 3:**
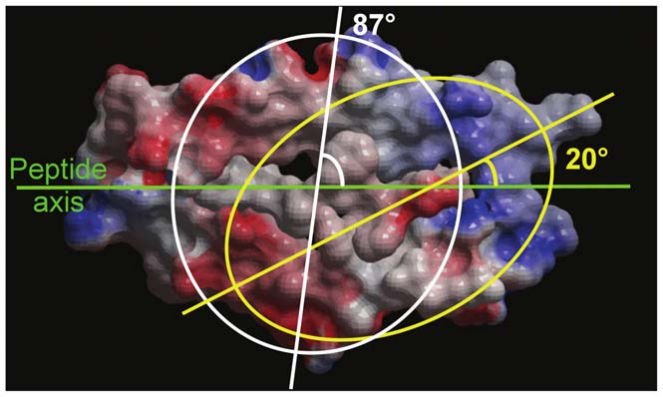
TR docking angle (θ) range computed using charge distribution on pMHC interfaces with reference to the axis of cognate peptide. Charges displayed on pMHC interface are located at an angle (θ) with respect to the axes of linear peptides (green), ranging from 20° (yellow ellipse) to 87° (white ellipse) (spanning 68°) over the entire dataset, which is similar to and overlaps the range of diagonal angles (50°; 22°–71°) for TR docking reported earlier [17].

**Figure 4:**
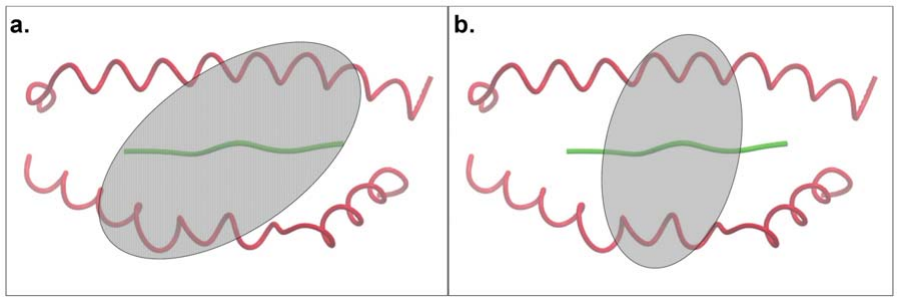
Relationship between θ and area covered by TR paratope on pMHC interface. **a**. Small θ value leading to a large interaction area compared to b. Large θ, resulting in a smaller paratope area. pMHC binding interface is shown as Cα trace with MHC helices in red and cognate peptide in green. Ellipses represent TR paratopes on pMHC, which are at distinct small and large θ with respect to the axis of bound peptides (angle calculation is shown previously in Figure 3). Shaded regions within the ellipses denote corresponding areas covered by TR paratopes. These areas clearly suggest large and small number of contacts that TR could make with pMHC in **a**. and **b**., respectively.

It has been reported earlier that diagonal angle of TR docking on pMHC varies between 22°–71° spanning a range of about 50° [17]. Charges displayed on MHC helices, when considered together, seem to present themselves at an angle. Utilizing the location of these charges, we have computed the corresponding TR docking angle (θ) on each pMHC interface (see Methods section for details). Our TR docking angle calculation results show that apart from the PDB structure 1ymm (θ of 112°; Table S1), whose diagonal TR docking angle (110°) has been reported to be of an unusually high value [44], θ varies between 20°–87° over the entire dataset (Figure 3), clearly overlapping the previously reported range of 22°–71° [17] and extending it in both directions. These results provide further evidence for docking of TR onto pMHC interface at an angle such that the TR appears almost “diagonally” [17] attached to the pMHC surface. θ for TR/pMHC-II structures was generally around 72° while for TR/pMHC-I complexes it was 42° on average. We note that when a TR docks onto pMHC interface with a low θ, the area covered by TR paratope on pMHC interface is greater due to the increased number of possible contacts between TR and pMHC interfaces (Figure 4a), therefore, implying that smaller the θ, stronger the binding interaction between TR and pMHC and *vice versa* (Figure 4b). This could possibly be one of the underlying reasons as to why a recent TR-like antibody designing study has yielded a Fab 3M4E5-based “Fab T1” antibody which gives a 20-fold affinity improvement compared to Fab 3M4E5 (PDB code 3hae; [45]) itself and exceeds the affinity of the original TR (1G4; PDB code 2bnr; [26]) by 1,000-fold, thereby, resulting in increased T cell cytotoxic activity [45]. The Fab 3M4E5 antibody (which itself has a 100-fold improvement in affinity compared to the original 1G4 TR [45]) binds the peptide/HLA-A*0201 complex (PDB code 3hae) at an angle of 40° [45] when compared to the diagonal TR docking angle of 69° (θ by our calculations is 39°) for the original 1G4 TR (PDB code 2bnr) [26], [45] and it makes more contacts with the pMHC compared to the 1G4 TR causing increased T cell cytotoxicity [45]. These additional interactions are between the A*0201 G-ALPHA2 residue A158 (IMGT 69) and the Fab 3M4E5 VH domain residues G56 & T58 (IMGT 63 and 65), A*0201 G-ALPHA2 residue Y159 (IMGT 70) and Fab 3M4E5 VH domain residue S57 (IMGT 64), A*0201 G-ALPHA2 domain residue T163 (IMGT 73) and Fab 3M4E5 VH domain residues G55 & S57 (IMGT 62 and 64), A*0201 G-ALPHA2 domain residues E166 & W177 (IMGT 76 and 77) and Fab 3M4E5 VH domain residue S54 (IMGT 59), which cause a change in the angle with which the antibody binds the pMHC complex [45], thereby supporting our hypothesis.

### BE is inversely proportional to θ

**Figure 5:**
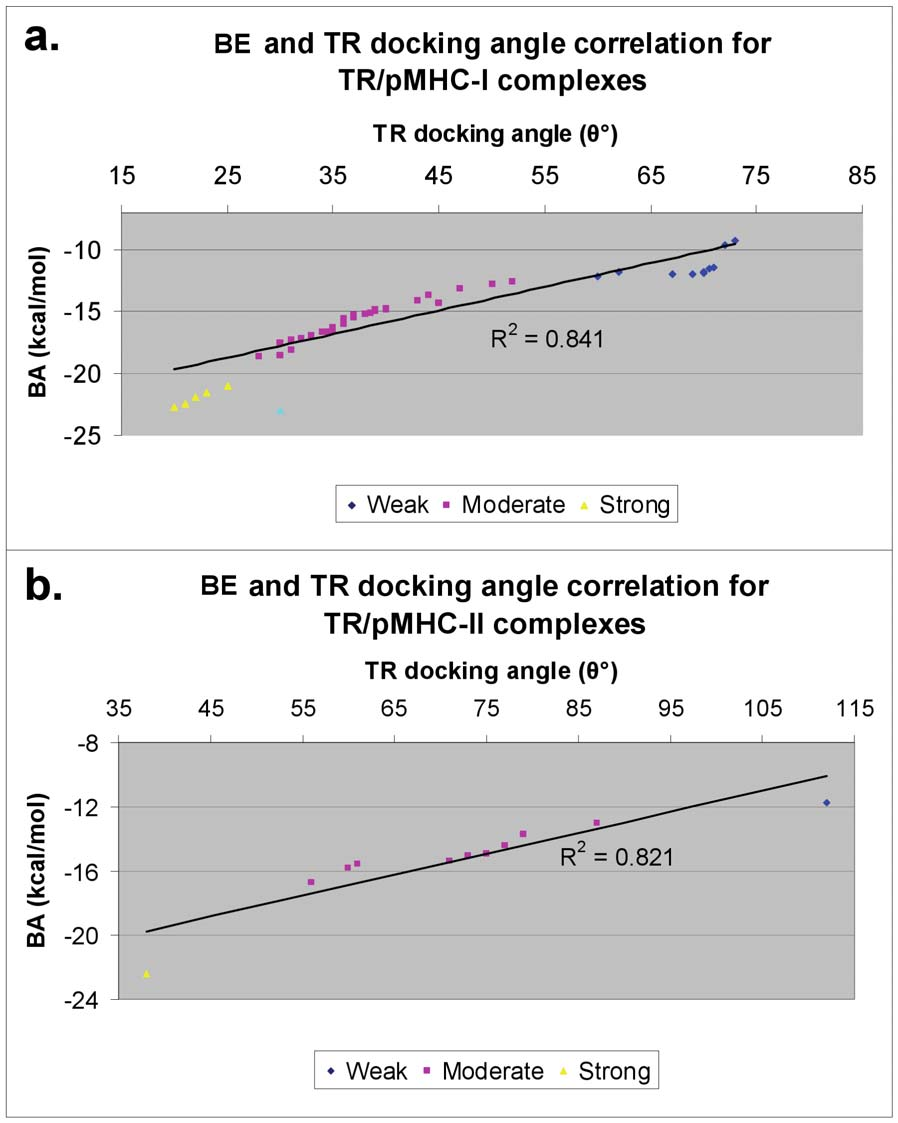
Correlation between BE and θ for a. pMHC-I agonists and b. pMHC-II complexes. The regression coefficients *r^2^ = *0.841 for pMHC-I agonists and *r^2^ = *0.821 for pMHC-II complexes are shown. The single outlier (PDB code 1lp9) in **a.** is highlighted in cyan.

Utilizing TR BE values computed for pMHC-I and pMHC-II weak-, moderate- and strong agonists and θ calculated using MSEP on their pMHC binding interfaces, we have established a significant correlation between BE and θ, as shown in Figure 5. Evidently, weak-agonists have a higher θ when compared to moderate-agonists and strong-agonists. Strong-agonists have the least θ amongst both TR/pMHC-I and TR/pMHC-II structures. This observation clearly highlights the significance of the derived correlation suggesting that for a given pMHC complex, TR BE is inversely proportional to θ and implying that, lower the θ stronger the binding between pMHC ligand and the respective TR and *vice versa*. Graphs in Figure 5 are explanatory of the above said correlation. Pearson correlation coefficient (r) between BE and θ for TR/pMHC-I complexes is 0.92 with a regression coefficient (*r^2^*) of 0.841. Similarly, for TR/pMHC-II complexes, Pearson correlation coefficient (r) is 0.91 and regression coefficient *r^2^ = *0.821. Interestingly, one TR/pMHC-I structure (1lp9; cyan in Figure 5a) seems to be an outlier from our correlation despite being classified as a strong-agonist. This was primarily owing to the collaborative contribution of the Vα CDR1, 2 and 3 loops which bind strongly to the MHC G-ALPHA2 residues 154–167 (IMGT 65–77) and MHC G-ALPHA1 residues 65–69 (IMGT 65–69) [46]. Comparatively, the binding exhibited by Vβ CDR1 which only binds to the peptide residue F6 (IMGT 6) and Vβ CDR2 loops that bind to MHC G-ALPHA1 residues 65–72 (IMGT 65–72), respectively, is weak with only Vβ CDR3 loops binding strongly to MHC G-ALPHA2 residues 146–155 (IMGT 58–66), resulting in an overall greater diagonal TR docking angle [46]. Therefore, the strong binding of Vα CDR1, 2, 3 and Vβ CDR3 loops with MHC G-ALPHA1 and G-ALPHA2 residues coupled with the tilt in the TR paratope due lack of interactions between Vβ CDR1 and MHC residues and weak interactions between Vβ CDR2 loops with MHC G-ALPHA1 resulted in our observations of the 1lp9 structure having an overall high TR/pMHC BE and a relatively higher θ value compared to other strong-agonists. Hence, this outlier was removed from our depicted correlation for TR/pMHC-I structures in Figure 5a. Upon inclusion of the outlier, the Pearson correlation coefficient (r) between BE and θ for TR/pMHC-I complexes decreases to 0.90 with a reduced regression coefficient (*r^2^*) of 0.808.

### TR paratope and pMHC epitope analyses reveal conserved positions

Residues on TR variable domains that contact the residues on pMHC interface are collectively referred to as “TR paratope”. Similarly, residues on pMHC interface that contact the residues on TR variable domains are collectively termed as “pMHC epitope”. Analyzing TR paratope and pMHC epitope across a wide dataset such as this is an important aspect in our quest to uncover the physicochemical basis of TR specificity and pMHC selectivity. Our results reaffirm the results of Garcia *et al*., [4] and Rudolf *et al*., [15] that there were no major conserved contacts observed between TR variable domains and pMHC interfaces over the entire dataset. However, we note that there are sets of pMHC ligands which have strikingly similar, even identical, patterns of interacting residues. Same is the case with TR variable domains which seem to fall into sets which show highly conserved patterns of interacting residues. These sets, along with MSEP based cluster dendograms (Fig. S1) and heat maps (Fig. S2) for pMHC interfaces obtained from our MSEP analysis, were used to cluster TR proteins (see Methods section for details). This characteristic was prominent in both TR/pMHC-I and TR/pMHC-II sequences.

**Figure 6:**
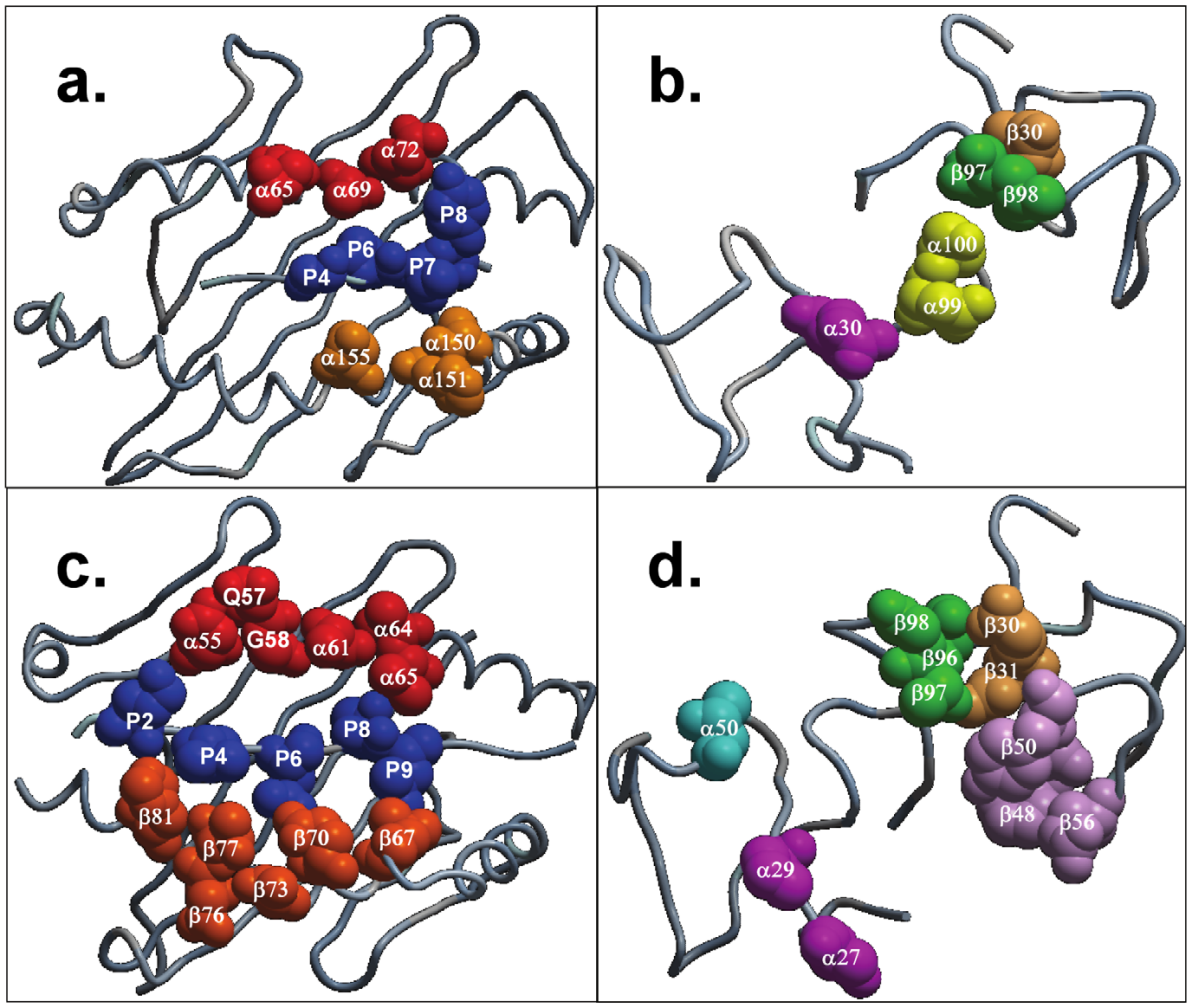
Residue conservation at pMHC and TR interfaces for a. pMHC-I ligands. b. pMHC-I binding TR. c. pMHC-II complexes and d. pMHC-II-binding TR. Conserved residue Q57 (IMGT 65) and mostly conserved residue G58 (IMGT 66) on G-ALPHA helix of pMHC-II interface in c are labelled. Conserved positions are labelled according to their chain locations on pMHC and TR interfaces. Highlighted in red are conserved positions, a conserved residue and a mostly conserved residue on G-ALPHA1 helix of pMHC-I and G-ALPHA helix of pMHC-II interfaces in **a.** and **c.**, respectively. Conserved positions on G-ALPHA2 helix of pMHC-I in **a.** are in gold. Residue positions on peptides are in blue and on G-BETA helix of pMHC-II in **c.** are in orange. Conserved residues and positions in **b.** and **d.** are coloured according to their CDR loops as follows: Vα CDR1: pink, CDR2: cyan, CDR3: yellow, Vβ CDR1: pale orange, CDR2: pale pink and CDR3: green. The colouring scheme used for CDR loops is the same used in Figure 2b. Protein backbones are represented as Cα trace in grey.

One, very significant and highly conserved contact was observed on all 11 pMHC-II interfaces. This residue was Gln (Q) 57 (IMGT 65), while Gly (G) 58 (IMGT 66) was mostly conserved on MHC G-ALPHA helix (labeled in Figure 6c). These residues are of utmost importance, as it could be this pair along with a few peptide residues that the TR variable domains could be looking for TR/pMHC complex formation in TR/pMHC-II structures. Amongst TR/pMHC-II complexes, these residues, perhaps serve as an alarm for TR signaling. Besides these conserved residues, we identified several conserved positions on the peptides, G-ALPHA1 and G-ALPHA2 MHC-I helices (Figure 6a), G-ALPHA and G-BETA MHC-II helices (Figure 6c), CDR1, CDR2 and CDR3 loops of respective pMHC-I and pMHC-II binding TR Vα and Vβ domains (Figure 6 b and d). These conserved residues and positions identified are listed in Table 1.

**Table 1:** List of conserved residues and positions.

MHC Class	Structural Location	Loop	Conserved Residues	Conserved Positions
I	MHC G-ALPHA1 helix	-	-	α65, α69 and α72
	MHC G-ALPHA2 helix	-	-	α150, α151 and α155
	Peptide	-	-	P4, P6, P7 and P8
	TR Vα	CDR1	-	α30
		CDR2	-	-
		CDR3	-	α99 and α100
	TR Vβ	CDR1	-	β30
		CDR2	-	-
		CDR3	-	β97 and β98
II	MHC G-ALPHA helix	-	Q57 and G58 (mostly conserved)	α61, α64 and α65
	MHC G-BETA helix	-	-	β67, β70, β73, β76, β77 and β81
	Peptide	-	-	P2, P4, P6, P8 and P9
	TR Vα	CDR1	-	α27, α29
		CDR2	-	α50
		CDR3	-	-
	TR Vβ	CDR1	-	β30 and β31
		CDR2	-	β48, β50 and β56
		CDR3	-	β96, β97 and β98

At this stage there are no absolutely conserved residues found in the interacting regions of TR/pMHC-I structures on the whole, but, as said above, there seems to be grouping and a definite pattern of conserved positions on interacting regions of both pMHC and TR, which present different combination of residues according to complementary MSEP displayed on corresponding interacting regions. Therefore, specificity of TR for one pMHC could possibly come from the specific pattern of interacting residues exhibited by that particular pMHC ligand at the above described conserved positions for both pMHC-I and pMHC-II. Based on our observations, we suggest that conserved residues along with residue variations at conserved positions form the basis of TR selectivity and specificity. Hence, these results, together with the common electrostatic rings seen on pMHC interfaces, explain the ability of a TR to survey many pMHC complexes before actually binding to one specific pMHC. Interestingly, number of conserved positions for TR/pMHC-I structures, are less compared to that of TR/pMHC-II structures. One fact that could be attributed to such a result is the small proportion of TR/pMHC-II structures (11) when compared to TR/pMHC-I (50) structures in the current data. Nevertheless, one could easily comprehend that with the increase in number of TR/pMHC-II structures, the number of conserved positions would eventually decrease.

Combining the results from our TR paratope, pMHC epitope and TR docking angle analyses, it is obvious that when a TR docks onto a pMHC binding interface with an overall small θ, the number of contacts between pMHC and TR are greater, thereby, increasing the area covered on pMHC interface by TR Vα and Vβ domains (TR paratope; Figure 4a), compared to the area covered when the TR docks with an overall large θ (Figure 4b), hence proving our earlier inference. This increase or decrease in number of contacts between pMHC and TR according to the decrease and increase in θ, respectively, has a direct consequence on BE between pMHC and TR as shown in the above correlation.

### TR grouping is allele and species dependent but TR specificity is peptide dependent

Calculation of MSEP similarities for all pMHC interfaces using webPIPSA server [47] and CLUSTALX [48] multiple sequence alignment of all TR paratopes and pMHC epitopes, have together provided us substantial evidence to define grouping (clustering) among TR proteins (see Methods section for details). These analyses formed the basis of our understanding of TR/pMHC binding and pMHC recognition similarities shown by TR proteins. webPIPSA uses the software R [49] for statistical computing and analytical grouping to produce a dendrogram (Fig. S1) and generate a heat map (Fig. S2). Table S1 portrays a clear clustering amongst TR proteins obtained by summarizing the results of webPIPSA analysis and multiple sequence alignment for TR paratopes and pMHC epitopes. By initial mapping of respective MHC alleles onto cluster dendograms in Figure S1, it was evident that similarities in MSEP displayed by pMHC interfaces were allele based.

Further investigation by mapping corresponding TR types (names for all TR proteins obtained from the literature) onto cluster dendograms alongside MHC alleles revealed that many TR proteins bind to same MHC allele which in turn is bound to different peptides (Table S1). This implies that TR specificity is perhaps primarily peptide dependent rather than completely allele dependent, shedding light on the impact of peptide properties in this significant immunological synapse, thus, further enforcing our earlier conclusion and weakening the “TR-MHC germline bias” theory. As seen, there were three clusters identified among pMHC-I binding TR proteins. Cluster I.1 comprises of six different types of TR proteins all of which are known to bind pMHC with murine MHC alleles. Cluster I.2 is made up of eight TR types which behave in a more diverse fashion by binding to pMHC with human alleles other than A*0201. Eight types of TR proteins which recognize pMHC-I with A*0201 allele fall under Cluster I.3. pMHC-II binding TR proteins were segregated into two distinct clusters, where, Cluster II.1 has five types of TR proteins which are associated with murine I-Au, I-Ag7 and I-Ak alleles and Cluster II.2 includes four TR types associated with human DR-alleles. These results are also noted to be species specific since all murine pMHC structures are clustered together implying that all TR types associated with murine MHC alleles are clustered together. This adds another dimension to this significant TR grouping system. It is worth noting that at the TR level the MHC supertype definitions do not apply.

Interestingly, there are multiple PDB structures for a single TR/pMHC complex, showing different TR binding angles, where we have tested the validity of our inverse relationship between calculated BE and θ. 2f54 and 2bnr (PDB code; bold in Table S1) form one such pair. Here, θ for 2f54 was computed to be 36° which is 3° smaller than that of 2bnr (39°). The calculated BE values for the two structures are −15.6 kcal/mol (2f54) and −14.9 kcal/mol (2bnr), respectively, which are inversely related to the θ values. These subtle changes in θ and BE are due to the underlying fact that the side chain of Q155 (IMGT 66) residue from MHC G-ALPHA2 domain forms a hydrogen bond with the side chain of TR Vα residue S51 (IMGT 58) in 2f54 [50] resulting in a well ordered Q155 (IMGT 66) side chain, when compared to its relatively disordered side chain orientation due to hydrogen bond formation with the side chain of TR Vα residue T95 (IMGT 109) in 2bnr [26]. Similarly, 2vlj, 2vlk and 1oga (bold and italics in Table S1) represent the same TR/pMHC complex, with different TR docking orientations. Compared to that of 1oga (69°; [17]), the diagonal TR docking angles for 2vlj and 2vlk are reported to be roughly up to 5° larger [51], whereas our computed θ values are 1° and 1.5° larger than both the diagonal TR docking angle and the computed θ value for 1oga (69°), respectively. Their respective calculated BE values are −11.7 kcal/mol (2vlj), −11.4 kcal/mol (2vlk) and −11.9 kcal/mol (1oga), which are in accord with our computed θ values and the diagonal TR docking angles reported. Yet again, the core residues involved in TR/pMHC interaction are conserved in all three of these structures and slight variations in θ and BE are a direct consequence of the subtle positional changes accommodated by the peripheral residues at the binding interface through regulations in their side chain conformations [51]. These are mainly MHC G-ALPHA1 residue Q72 (IMGT 72), MHC G-ALPHA2 domain residue Q155 (IMGT 66) and the TR Vβ residue I53 (IMGT 58) [51].

## Discussion

We have analyzed available TR/pMHC structures using a number of physicochemical characteristics to understand any basic differences between pMHC-I and pMHC-II interactions with TR. The avidity of TR/pMHC interaction has been classified as weak-, moderate-, and strong-, based on the BE values that were computed for pMHC and TR binding interfaces. Using BE as a discriminator between weak-, moderate- and strong-agonists will add value to prediction methods enabling them to successfully predict true T cell epitopes or strong-agonists that are highly likely to initiate T cell response. Also, it would be interesting to decompose BE into electrostatic and van der Waals components to get an insight into the energetic contributions and correlate these with the differing amino acids at the TR and pMHC interfaces. We have also proposed a novel and rational approach to computing θ value by mapping charged rings formed from MSEP on the pMHC interface. Here, we note from literature that, although for some TR/pMHC crystal structures the entire TR paratope is used to calculate the diagonal TR docking angle [17], using the central mass of TR Vα and Vβ domains as a reference to draw an axis [46], [52] that cuts the cognate peptide axis at an angle (generally much greater than the angle obtained by using the entire paratope) appears to be the common practice of diagonal TR docking angle calculation for most crystal structures. Hence, the fact that we employ TR paratope, pMHC epitope and MSEP at pMHC interfaces to procure the θ values, could be the fundamental reason for our θ values being extremely close or fairly distant to the diagonal TR docking angles reported for some structures (Table S1). Results from our MSEP analysis explain the common TR docking geometry on pMHC interface, seen in all TR/pMHC structures. None of the structures available to us for analysis has a glycan molecule at or near the TR/pMHC interface. However, some of these molecules have a glycan shield around them which may also contribute towards docking by excluding certain modes of binding and helping in orientation of TR [53]. This is a possible complexity that needs to be factored in as more data becomes available. Using MSEP in epitope prediction methods could further accelerate the progress of structure-based prediction techniques besides minimizing false positives and true negatives from actual agonistic peptides in a given set of peptide antigens. We have reported a strong correlation between BE values and θ across the entire dataset which solves the first query addressed in this manuscript (described earlier in Introduction section). Analysis of TR paratopes and pMHC epitopes revealed that although there are no absolutely conserved residues found in interacting regions of both TR and pMHC ligands, there are vital conserved positions on both interfaces across TR/pMHC-I and TR/pMHC-II structures that could have fundamental implication for peptide vaccine design. Identification of conserved residues/positions on pMHC and TR interacting regions provides clues to the positional specificity of TR proteins. Furthermore, we have clustered TR proteins based on their binding site similarities, pMHC recognition similarities and similarities in MSEP on their respective interacting pMHC interfaces, to dissect TR/pMHC binding requirements. MSEP similarity calculation at the pMHC interface together with TR paratope and pMHC epitope analyses have thus given us enough evidence to suggest a weakening of “germline bias” theory over a larger dataset and highlight the significant role played by the peptide in determining TR specificity, thereby, answering our second question (see Introduction section for details).

Based on our findings, we wish to propose a mechanism for TR/pMHC binding and TR activation which explains the phenomenon of pMHC recognition by TR and TR specificity simultaneously. We suggest that, after peptide binding to MHC, many similar pMHC complexes are presented on the cell surface which exhibit similar charged rings of MSEP (explained earlier in the results of our TR and pMHC interface MSEP analysis) thereby signalling or attracting the TR towards them through long-range electrostatic steering. Due to their electrostatic similarity, the TR actually surveys many pMHC complexes. This is possible by temporary interactions between the rings of charged residues displayed on MHC helices and on CDR1 and CDR2 loops of TR Vα and Vβ domains. This phenomenon is followed by the recognition of specific arrangements of pMHC residues (at conserved positions) by CDR3 loops. Once this recognition occurs, the TR localizes itself on the pMHC such that the half-life of TR/pMHC complex is sufficiently stabilized for T cell activation. Therefore, the entire process of pMHC recognition and TR signalling is possibly governed by two factors, the electrostatic ring displayed by pMHC interface and a specific arrangement of residues presented by pMHC.

From our extensive studies on TR/pMHC interactions we have defined structural features that can be analyzed as parameters governing TR/pMHC complex formation relevant for immune system activation. These parameters are MSEP of TR and pMHC interfaces and TR docking angle (θ), which, when coupled with the knowledge of specific arrangement of residues at conserved positions on TR and pMHC interfaces, could be used as discriminants for *in* silico identification of strong-agonistic pMHC complexes. Results of these analyses could be used to develop and/or enhance methods to successfully predict T cell epitopes in accordance with their MHC and TR binding specificities. This could greatly improve the efficacy of T cell epitope prediction models in separating true T cell epitopes from a large number of predicted MHC-binding peptides. This kind of structure-based screening helps overcome the barriers of insufficient training data and lack of peptide binding motifs, especially for MHC-II alleles, thereby cutting down the lead time involved in experimental vaccine development methods, resulting in production of effective and highly specific peptide vaccines with a wide population coverage. Our results will facilitate the rational development of peptide vaccines, capable of eliciting T cell response, for immunotherapies to protect against or combat infectious, autoimmune, allergic and graft *vs*. host diseases.

## Methods

### Data

The data used in this study comprises of 61 non-redundant TR/pMHC structures from the MPID-T2 database (http://biolinfo.org/mpid-t2) [8], which were originally obtained from the Protein Data Bank (PDB) [9] and verified with the IMGT/3Dstructure-DB (http://www.imgt.org/3Dstructure-DB/) database [10], [11]. The PDB structure 2icw was not included in this study as it has a superantigen between the TR and the pMHC which prevents actual TR/pMHC interaction by mediating the TR/pMHC binding [54]. Out of the 61 structures, 50 were MHC-I complexes spanning 9 alleles from human (7) and mouse (2) and 11 MHC-II complexes spanning 7 alleles, again from human (4) and mouse (3). When there is more than one structure with the same peptide sequence, MHC allele and TR type, mutations in the MHC α (I-ALPHA) chain (MHC-I), TR Vα and Vβ CDR2 & 3 loops and the degree of tilt or relative change (compared to the first structure with similar TR type, MHC allele and peptide sequence in Table S1) in θ were taken into account as primary criteria to consider the structures non-redundant. Coordinates for truncated versions of the X-ray structures, encompassing single structural complexes of the pMHC binding interfaces and the variable domains of the TR were extracted for TR paratope, pMHC epitope analyses and MSEP calculations.

### BE calculation

The interaction of most ligands with their binding sites can be characterized in terms of binding free energy or binding energy (BE). In general, high energy TR/pMHC binding results from greater intermolecular force between the pMHC and its TR while low energy ligand binding involves less intermolecular force between the pMHC and its TR. High energy binding involves a longer residence time for the TR on its respective pMHC than in the case of low energy binding. High energy binding of pMHC to a TR is often physiologically important as some of the BE can be used to cause a conformational change in the TR, resulting in a physiological response or T cell response [55], [56]. Since BE is also referred to as binding free energy, the most negative value is considered the best. In literature, BE (*ΔG*) is usually derived from the binding constants of the interaction such as *K_d_* and *K_a_*.

The general thermodynamic formulae used are as follows:
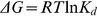
(1)

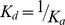
(2)where *K_d_* is the dissociation constant, *R* is the universal gas constant, *T* is the absolute temperature and *K_a_* is the association constant. BE values between the pMHC and TR for all TR/pMHC structures were calculated using the program DCOMPLEX [57], which uses DFIRE-based potentials [58]. The program first calculates the total atom-atom potential of mean force, *G*, for each structure, which is given by:
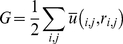
(3)where *ū* is the atom-atom potential of mean force between two atoms, *i* and *j* that are a distance *r* apart, the summation is over atomic pairs that are not in the same residue and a factor of ½ is used to avoid double-counting of residue-residue and atom-atom interactions [57].

The binding free energy between two interacting proteins *A* and *B* can also be obtained by using:
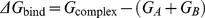
(4)where *A* and *B* are considered as two rigid bodies whose interface residues contribute most to *ΔG*_bind_ [57]. Therefore, the final equation used by DCOMPLEX [57] to calculate BE is as follows:
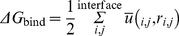
(5)


DCOMPLEX provides an overall BE, without details of specific components for electrostatic, van der Waals, hydrophobic and entropic terms.

### MSEP similarity calculation

MSEP in proteins is a result of charged side chains of the amino acid residues and bound ions. These potentials play a vital role in protein folding, stability, enzyme catalysis and specific protein-protein recognitions. MSEP similarity between any two protein molecules is a measure of the similarity in their composition of charged residues. Interactions between the TR and pMHC in all the structures depend vastly on the charges that the binding site on the pMHC displays. Thus, the web server webPIPSA [47] was used to calculate the MSEP and compare the electrostatic interaction properties of only the pMHC binding interfaces in all the structures. The algorithm begins with calculation of the protein MSEP and then calculates similarity indices for all pairs of proteins based on the electrostatic similarity. The similarity indices are then converted to electrostatic distances which are then displayed as a colour coded matrix called as the heat map (Fig. S2) and as a tree or a cluster dendogram (Fig. S1). These cluster dendograms and heat maps were consequently used for TR clustering (described below). Structural models of only the pMHC interfaces were used for this analysis. ICM [59], [60] was then used to visually analyse the electrostatic images of all the structures.

### Calculation of TR docking angles (θ)

Similarly, we generated and visualized electrostatic images of the TR binding interfaces (Vα and Vβ domains). The respective pMHC and TR interfaces were then matched for complementarities of charges and the corresponding charges were numbered accordingly on both the interfaces (Figure 2). These charged residues were cross verified with the list of pMHC and TR interacting residues collated for TR paratope and pMHC epitope residue conservation analyses. The charged residues missing from these lists were omitted and the charges were renumbered for consistency in results. A line was drawn which connects the numbers on each of the pMHC interfaces using ICM [59], [60]. Once connected, the numbers on a given pMHC interface formed an ellipsoidal shape, which determines the TR paratope on the pMHC (Figure 3). These ellipses were noticed to be at a certain angle with respect to the Cα backbone axes of the respective cognate peptides across the entire dataset. Finally, straight lines were drawn diagonally across the ellipses which cut the axes of the bound peptides at a given angle (Figure 3). These angles were measured using ICM [59], [60] and are called TR docking angle (θ) on the pMHC interfaces (Figure 3).

### TR paratope and pMHC epitope residue conservation analyses

These analyses required us to manually extrapolate and list the interacting residues of the pMHC and TR for each structure either from the literature or by using ICM [59], [60] computer program. CLUSTALX [48] was later used to perform multiple sequence alignment in the hope of identifying any conserved patterns in the interacting residues of pMHC and TR interfaces.

### TR grouping

Initially, the sets of pMHC and TR interfaces, obtained from our TR paratope and pMHC epitope residue conservation analyses, showing similar pattern of interacting residues (mentioned earlier in the Results section), were matched against the cluster dendograms (Fig. S1) and heat maps (Fig. S2), to verify if the structures that display the sets observed in residue conservation analyses, are present within distinct clusters of pMHC complexes (Fig. S1 and S2). After this confirmation, the respective MHC alleles and corresponding TR types were mapped onto the cluster dendograms which clearly indicated the grouping (clustering) amongst the TR molecules based on similarities in their binding site, pMHC recognition properties and MSEP displayed on their respective interacting pMHC interfaces.

## Supporting Information

Table S1 **Grouping of TR proteins.**
Mutations in MHC α (I-ALPHA) chain and TR Vβ domain (MHC-I; TR Cluster I.2 and I.3), TR mutant names and the degree of tilt or relative change (compared to the first structure with similar TR type, MHC allele and peptide sequence) in θ are mentioned in parentheses (see Methods section for details).
(PDF)

Figure S1 **Cluster dendograms for all pMHC interfaces based on their MSEP similarities. **a. pMHC-I complexes clustered into three distinct clusters. b. pMHC-II ligands clustered into two distinct clusters.
Each pMHC interface is denoted by its corresponding PDB code. Every pMHC is mapped onto its respective MHC allele and the interacting TR type (TR name). This clearly indicates the clustering amongst the TR proteins. The three distinct clusters of pMHC-I binding TR proteins are coloured yellow: cluster I.1, green: cluster I.2 and orange: cluster I.3. The two clusters amongst pMHC-II binding TR proteins are highlighted in light blue: cluster II.1 and lavender: cluster II.2. TR grouping (clustering) is in accordance with Table S1.
(PDF)

Figure S2 **Heat maps for all pMHC interfaces based on the calculated MSEP values depicted as a colour coded matrix showing clustering amongst pMHC complexes in a reverse order as compared to the cluster dendograms in Figure S1. **a. pMHC-I complexes clustered into three. b. pMHC-II structures in two distinct clusters.
Each pMHC interface is again denoted by its corresponding PDB code. Inset, are the legends showing the color key used to create heat matrices and the MSEP value ranges for pMHC interfaces. Also shown is the formula used to calculate electrostatic distances for clustering.
(PDF)
