# Supplementary material for: Understanding TR Binding to pMHC Complexes: How Does a TR Scan Many pMHC Complexes yet Preferentially Bind to One
Source: PLoS One. 2011 Feb 22;6(2):e17194. doi: 10.1371/journal.pone.0017194 (PMC3043089; doi:10.1371/journal.pone.0017194)
Supplement: Table S1 — Grouping of TR proteins. Mutations in MHC α (I-ALPHA) chain and TR Vβ domain (MHC-I; TR Cluster I.2 and I.3), TR mutant names and the degree of tilt or relative change (compared to the first structure with similar TR type, MHC allele and peptide sequence) in θ are mentioned in parentheses (see Methods section for details). (PDF) [file pone.0017194.s001.pdf]

Supplementary Table S1

Understanding TR binding to pMHC complexes: how does the TR scan many pMHC molecules yet preferentially bind to one

Javed M. Khan and Shoba Ranganathan

Table S1. Grouping of TR proteins.

Mutations in MHC α (I-ALPHA) chain and TR Vβ domain (MHC-I; TR Cluster I.2 and I.3), TR mutant names and the degree of tilt or relative change (compared to the first structure with similar TR type, MHC allele and peptide sequence) in θ are mentioned in parentheses (see Methods section for details).

S.No.	PDB code	Species	Peptide Sequence	MHC Allele	BE (kcal/mol)	Experimental binding affinity (K_d in μM)	θ (°)	Diagonal TR docking angle (°) from literature	TR Type (TR name)	TR Cluster
**TR/pMHC-I**										
1	2oi9	Murine	QLSPFPFDL	H2-Ld	-13.13	-	47	-	2C	I.1
2	1g6r	Murine	SIYRYYGL	H2-Kb	-12.57	-	52	44 [46]	2C	
3	1mwa	Murine	EQYKFYSV	H2-Kbm3	-14.12	-	43	-	2C	
4	2ckb	Murine	EQYKFYSV	H2-Kb	-11.78	10	62	22 [17]	2C	
5	1fo0	Murine	INFDFNTI	H2-Kb	-9.61	2.6	72	58 [46]	BM3.3	
6	1nam	Murine	RGYVYQGL	H2-Kb	-11.99	114	67	-	BM3.3	
7	2ol3	Murine	SQYYYNSL	H2-Kb	-11.47	112	71	-	BM3.3	
8	2e7l	Murine	QLSPFPFDL	H2-Ld	-13.73	2 [23]	44	44	M6	
9	3e2h	Murine	QLSPFPFDL	H2-Ld	-12.16	4.6	60	-	M67	
10	3e3q	Murine	QLSPFPFDL	H2-Ld	-12.76	11.6×10^{-2}	50	-	M13	
11	1kj2	Murine	KVITFIDL	H2-Kb	-15.30	-	37	-	KB5-C20	

S.No.	PDB code	Species	Peptide Sequence	MHC Allele	BE (kcal/mol)	Experimental binding affinity (K_d in μM)	θ (°)	Diagonal TR docking angle (°) from literature	TR Type (TR name)	TR Cluster
**TR/pMHC-I**										
12	1w72	Human	EADPTGHSY	A*0101	-9.26	1.4×10^{-2}	73	-	FAB – HYB3	I.2
13	1mi5	Human	FLRGRAYGL	B*0801	-17.19	-	32	-	LC13	
14	3kpr	Human	EEYLKAWTF	B*4405	-21.88	1.54	22	-	LC13	
15	3kps	Human	EEYLQAFTY	B*4405	-21.52	49	23	-	LC13	
16	2esv	Human	VMAPRTLIL	E*0101	-12.79	30.2	50	50	KK50.4	
17	2nx5	Human	EPLPQGQLTAY	B*3501	-14.76	-	40	-	ELS4	
18	2ak4	Human	LPEPLPQGQLTAY	B*3508	-14.83	9.9	40	-	SB27	
19	3kxf	Human	LPEPLPQGQLTAY	B*3508 (Q65A, T69A, Q155A)	-18.70	-	28	-	SB27	
20	3ffc	Human	FLRGRAYGL	B*0801	-18.60	8.9	30	58	CF34	
21	3dxa	Human	EENLLDFVRF	B*4405	-18.10	0.3	31	80	DM1	
22	3mv7	Human	HPVGEADYFEY	B*3501	-16.96	-	33	-	TK3	
23	3mv8	Human	HPVGEADYFEY	B*3501	-16.68	-	34	-	TK3 (Vβ Q55H)	
24	3mv9	Human	HPVGEADYFEY	B*3501	-16.36	-	35	-	TK3 (Vβ Q55A)	
25	1bd2	Human	LLFGYPVYV	A*0201	-14.33	-	45	52 [17]	B7	I.3
26	1lp9	Human	ALWGFFPVL	A*0201	-22.97	10	30	89	AHIII 12.2	
27	2uwe	Human	ALWGFFPVL	A*0201 (T163A)	-22.44	4.7	21	-	AHIII 12.2	

S.No.	PDB code	Species	Peptide Sequence	MHC Allele	BE (kcal/mol)	Experimental binding affinity (K_d in μM)	θ (°)	Diagonal TR docking angle (°) from literature	TR Type (TR name)	TR Cluster
**TR/pMHC-I**										
28	2j8u	Human	ALWGFFPVL	A*0201 (K66A)	-21.01	31.8	25	-	AHIII 12.2	I.3 (contd.)
29	2jcc	Human	ALWGFFPVL	A*0201 (W167A)	-22.74	15.4	20	-	AHIII 12.2	
30	1ao7	Human	LLFGYPVYV	A*0201	-17.20	-	32	37 [17]	A6	
31	1qrn	Human	LLFGYAVYV	A*0201	-16.59	100	35	-	A6	
32	1qse	Human	LLFGYPRYV	A*0201	-17.28	8	31	-	A6	
33	1qsf	Human	LLFGYPVAV	A*0201	-14.99	-	39	-	A6	
34	2gj6	Human	LLFGKPVYV	A*0201	-15.49	160	37	-	A6	
35	3d39	Human	LLFGFPVYV (Y5{4-fluoroPhenylalanine})	A*0201	-15.16	0.64	38.5	-	A6	
36	3d3v	Human	LLFGFPVYV (Y5{3,4-difluoroPhenylalanine})	A*0201	-15.40	0.46	37	-	A6	
37	3h9s	Human	MLWGYLQYV	A*0201	-14.91	-	39	-	A6	
38	**2bnr**	Human	SLLMWITQC	A*0201	-14.93	13.3	39	69	1G4	
39	**2f54**	Human	SLLMWITQC	A*0201	-15.59	15	36	-	1G4 (-3°)	
40	2f53	Human	SLLMWITQC	A*0201	-16.06	1×10^{-3}	36	-	1G4 (c49c50)	
41	2p5e	Human	SLLMWITQC	A*0201	-16.08	48×10^{-6}	36	-	1G4 (c58c61)	
42	2p5w	Human	SLLMWITQC	A*0201	-16.69	-	34	-	1G4 (c58c62)	
43	2pye	Human	SLLMWITQC	A*0201	-15.21	8.16×10^{-2}	38	-	1G4 (c5c1)	
44	2bnq	Human	SLLMWITQV	A*0201	-15.23	5	38	68.1	1G4	

S.No.	PDB code	Species	Peptide Sequence	MHC Allele	BE (kcal/mol)	Experimental binding affinity (K_d in μM)	θ (°)	Diagonal TR docking angle (°) from literature	TR Type (TR name)	TR Cluster
**TR/pMHC-I**										
45	***1oga***	Human	GILGFVFTL	A*0201	-11.99	-	69	69	Vβ17 Vα10.2	I.3 (contd.)
46	***2vlj***	Human	GILGFVFTL	A*0201	-11.77	5.2	70	-	Vβ17 Vα10.2 (+5°)	
47	***2vlk***	Human	GILGFVFTL	A*0201	-11.48	5.2	70.5	-	Vβ17 Vα10.2 (+10°)	
48	2vlr	Human	GILGFVFTL	A*0201	-11.88	4.9	70	-	Vβ17 Vα10.2 (Vβ S99A)	
49	3gsn	Human	NLVPMVATV	A*0201	-16.66	27.7	34.5	35	RA14	
50	3hg1	Human	ELAGIGILTV	A*0201	-17.58	18	30	35	MEL5	
**TR/pMHC-II**										
1	1u3h	Murine	SRGGASQYRPSQ	I-Au	-15.06	-	73	-	172.10 Vβ8.2	II.1
2	2z31	Murine	RGGASQYRPSQ	I-Au	-13.01	25.3	87	-	cl 19 Vβ8.2	
3	2pxy	Murine	RGGASQYRPSQ	I-Au	-14.47	25.4	77	-	1934.4 Vβ8.2	
4	3mbe	Murine	GAMKRHGLDNYRGYSLGN	I-Ag7	-22.42	16.2×10^{-2}	38	48	21.30	
5	1d9k	Murine	GNSHRGAIEWEGIESG	I-Ak	-15.42	-	71	80	D10	
6	1j8h	Human	PKYVKQNTLKLAT	DRB1*0401	-16.76	-	56	-	HA1.7	II.2
7	1fyt	Human	PKYVKQNTLKLAT	DRB1*0101	-15.87	-	60	70	HA1.7	
8	1zgl	Human	VHFFKNIVTPRTPG	DRA*0101	-15.59	-	61	47	3A6	
9	2iam	Human	GELIGILNAAKVPAD	DRB1*0101	-13.72	-	79	71	E8	
10	2ian	Human	GELIGTLNAAKVPAD	DRB1*0101	-14.96	-	75	71	E8	
11	1ymm	Human	ENPVVHFFKNIVTP	DRB1*1501	-11.76	-	112	110	Ob.1A12	
