# Supplementary material for: Understanding TR Binding to pMHC Complexes: How Does a TR Scan Many pMHC Complexes yet Preferentially Bind to One
Source: PLoS One. 2011 Feb 22;6(2):e17194. doi: 10.1371/journal.pone.0017194 (PMC3043089; doi:10.1371/journal.pone.0017194)
Supplement: Figure S1 — Cluster dendograms for all pMHC interfaces based on their MSEP similarities. a. pMHC-I complexes clustered into three distinct clusters. b. pMHC-II ligands clustered into two distinct clusters. Each pMHC interface is denoted by its corresponding PDB code. Every pMHC is mapped onto its respective MHC allele and the interacting TR type (TR name). This clearly indicates the clustering amongst the TR proteins. The three distinct clusters of pMHC-I binding TR proteins are coloured yellow: cluster I.1, green: cluster I.2 and orange: cluster I.3. The two clusters amongst pMHC-II binding TR proteins are highlighted in light blue: cluster II.1 and lavender: cluster II.2. TR grouping (clustering) is in accordance with Table S1. (PDF) [file pone.0017194.s002.pdf]

Supplementary Figure S1

Understanding TR binding to pMHC complexes: how does the TR scan many pMHC molecules yet preferentially bind to one

Javed M. Khan and Shoba Ranganathan

a.

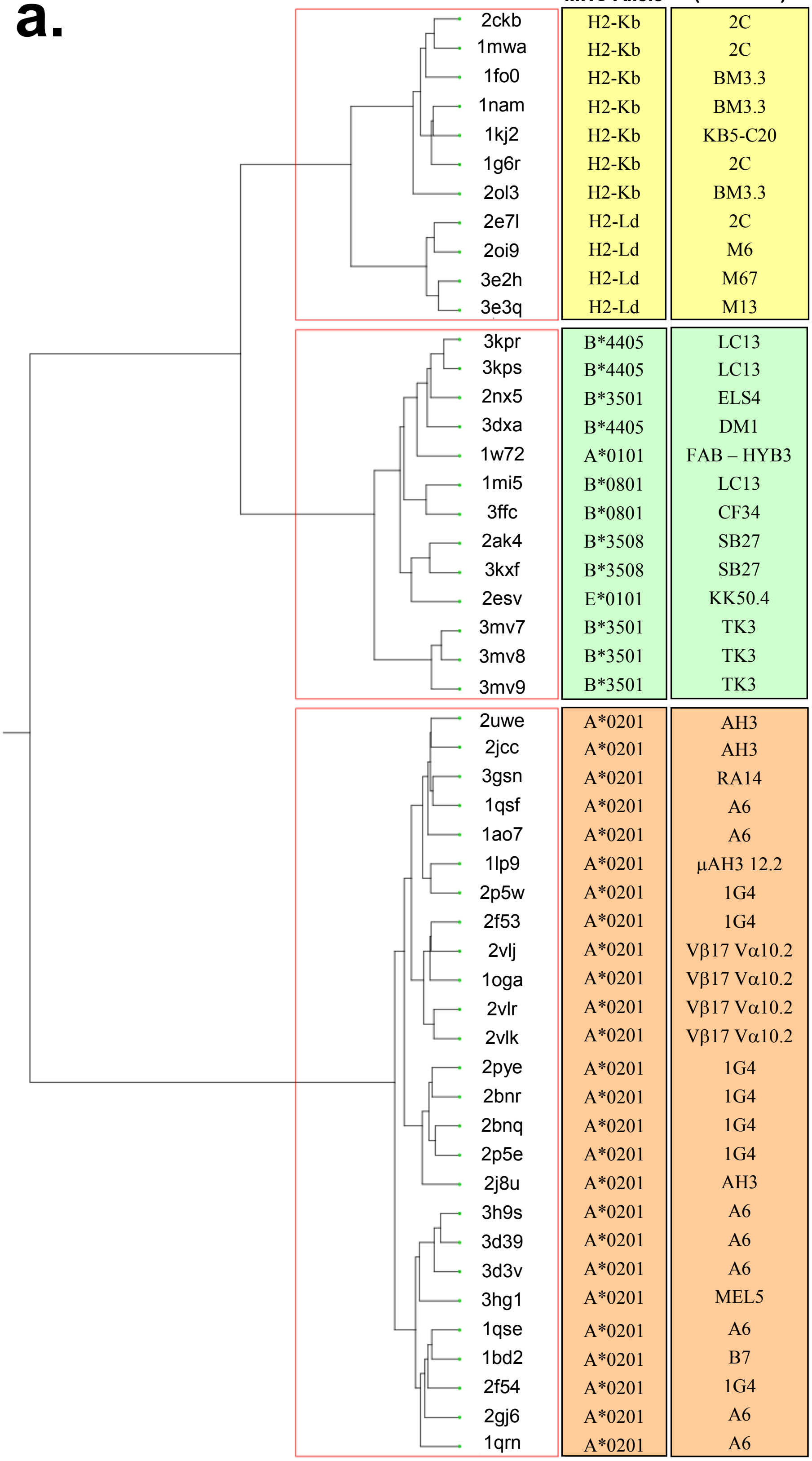

	MHC Allele	TR Type (TR name)
2ckb	H2-Kb	2C
1mwa	H2-Kb	2C
1fo0	H2-Kb	BM3.3
1nam	H2-Kb	BM3.3
1kj2	H2-Kb	KB5-C20
1g6r	H2-Kb	2C
2ol3	H2-Kb	BM3.3
2e7l	H2-Ld	2C
2oi9	H2-Ld	M6
3e2h	H2-Ld	M67
3e3q	H2-Ld	M13
3kpr	B*4405	LC13
3kps	B*4405	LC13
2nx5	B*3501	ELS4
3dxa	B*4405	DM1
1w72	A*0101	FAB – HYB3
1mi5	B*0801	LC13
3ffc	B*0801	CF34
2ak4	B*3508	SB27
3kxf	B*3508	SB27
2esv	E*0101	KK50.4
3mv7	B*3501	TK3
3mv8	B*3501	TK3
3mv9	B*3501	TK3
2uwe	A*0201	AH3
2jcc	A*0201	AH3
3gsn	A*0201	RA14
1qsf	A*0201	A6
1ao7	A*0201	A6
1lp9	A*0201	μAH3 12.2
2p5w	A*0201	1G4
2f53	A*0201	1G4
2vlj	A*0201	Vβ17 Vα10.2
1oga	A*0201	Vβ17 Vα10.2
2vlr	A*0201	Vβ17 Vα10.2
2vlk	A*0201	Vβ17 Vα10.2
2pye	A*0201	1G4
2bnr	A*0201	1G4
2bnq	A*0201	1G4
2p5e	A*0201	1G4
2j8u	A*0201	AH3
3h9s	A*0201	A6
3d39	A*0201	A6
3d3v	A*0201	A6
3hg1	A*0201	MEL5
1qse	A*0201	A6
1bd2	A*0201	B7
2f54	A*0201	1G4
2gj6	A*0201	A6
1qrn	A*0201	A6

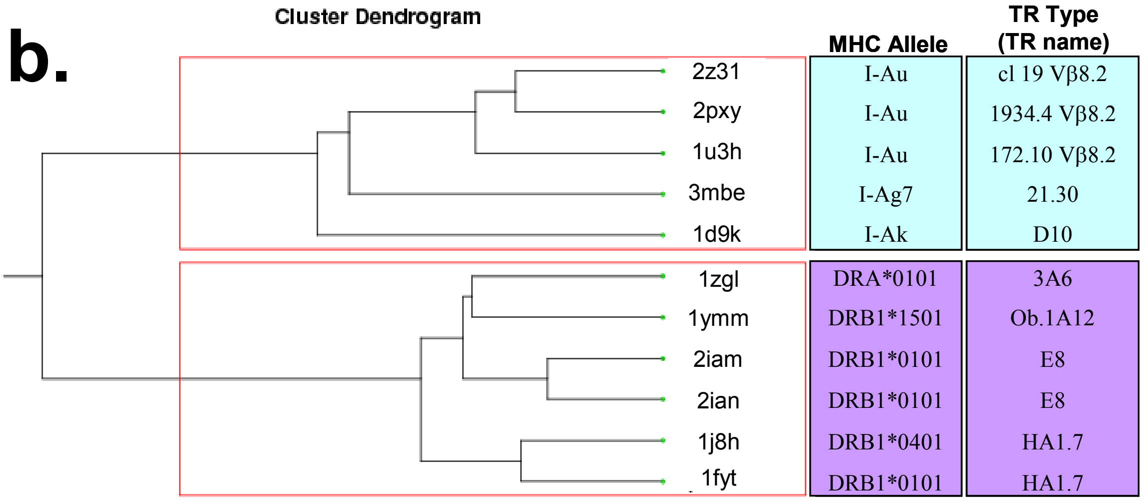

Figure S1. Cluster dendograms for all pMHC interfaces based on their MSEP similarities. a. pMHC-I complexes clustered into three distinct clusters. b. pMHC-II ligands clustered into two distinct clusters. Each pMHC interface is denoted by its corresponding PDB code. Every pMHC is mapped onto its respective MHC allele and the interacting TR type (TR name). This clearly indicates the clustering amongst the TR proteins. The three distinct clusters of pMHC-I binding TR proteins are coloured yellow – cluster I.1, green – cluster I.2 and orange – cluster I.3. The two clusters amongst pMHC-II binding TR proteins are highlighted in light blue – cluster II.1 and lavender – cluster II.2. TR grouping (clustering) is in accordance with Table 1.
