# Supplementary material for: Understanding TR Binding to pMHC Complexes: How Does a TR Scan Many pMHC Complexes yet Preferentially Bind to One
Source: PLoS One. 2011 Feb 22;6(2):e17194. doi: 10.1371/journal.pone.0017194 (PMC3043089; doi:10.1371/journal.pone.0017194)
Supplement: Figure S2 — Heat maps for all pMHC interfaces based on the calculated MSEP values depicted as a colour coded matrix showing clustering amongst pMHC complexes in a reverse order as compared to the cluster dendograms in Figure S1. a. pMHC-I complexes clustered into three. b. pMHC-II structures in two distinct clusters. Each pMHC interface is again denoted by its corresponding PDB code. Inset, are the legends showing the color key used to create heat matrices and the MSEP value ranges for pMHC interfaces. Also shown is the formula used to calculate electrostatic distances for clustering. (PDF) [file pone.0017194.s003.pdf]

Supplementary Figure S2

Understanding TR binding to pMHC complexes: how does the TR scan many pMHC molecules yet preferentially bind to one

Javed M. Khan and Shoba Ranganathan

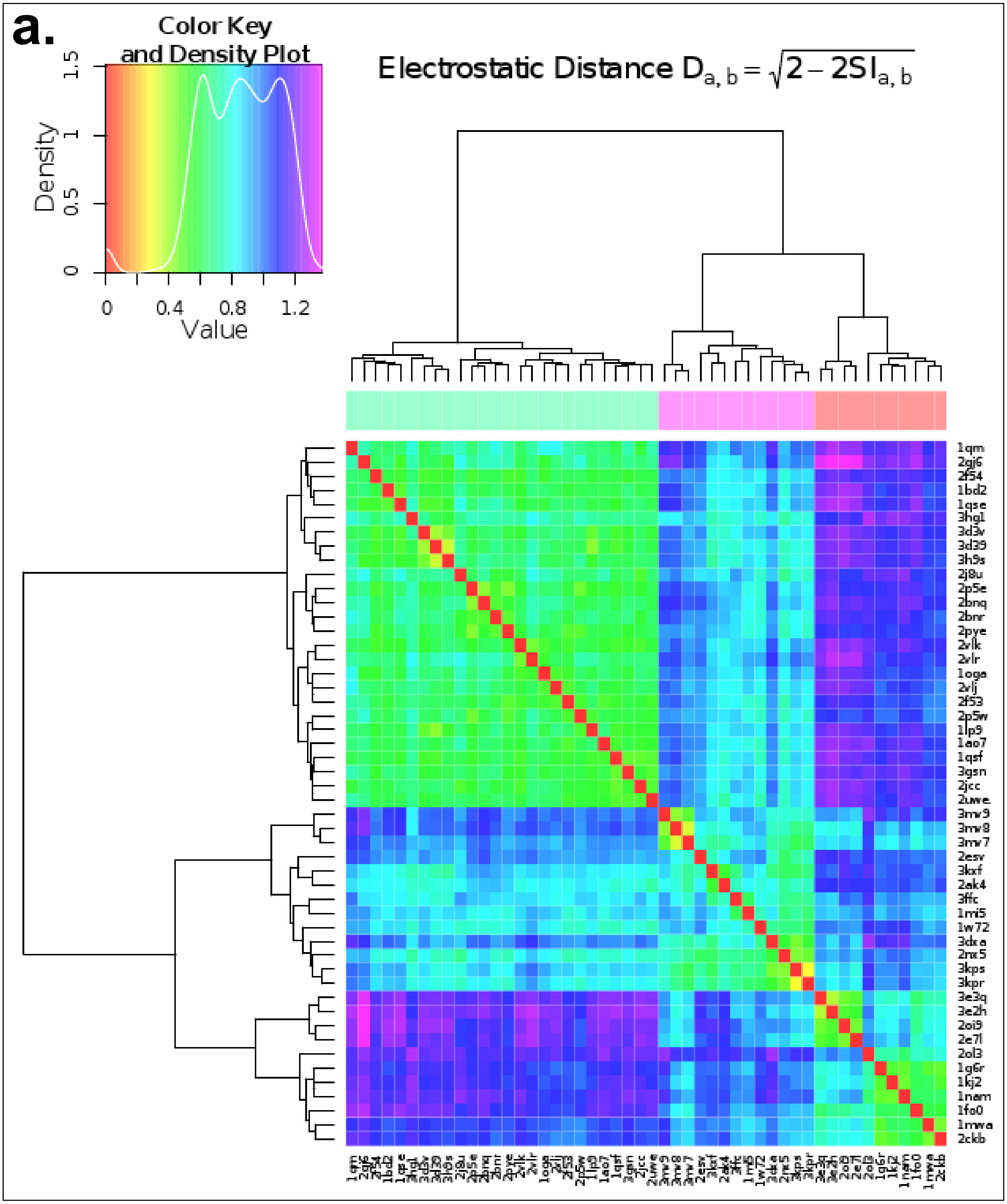

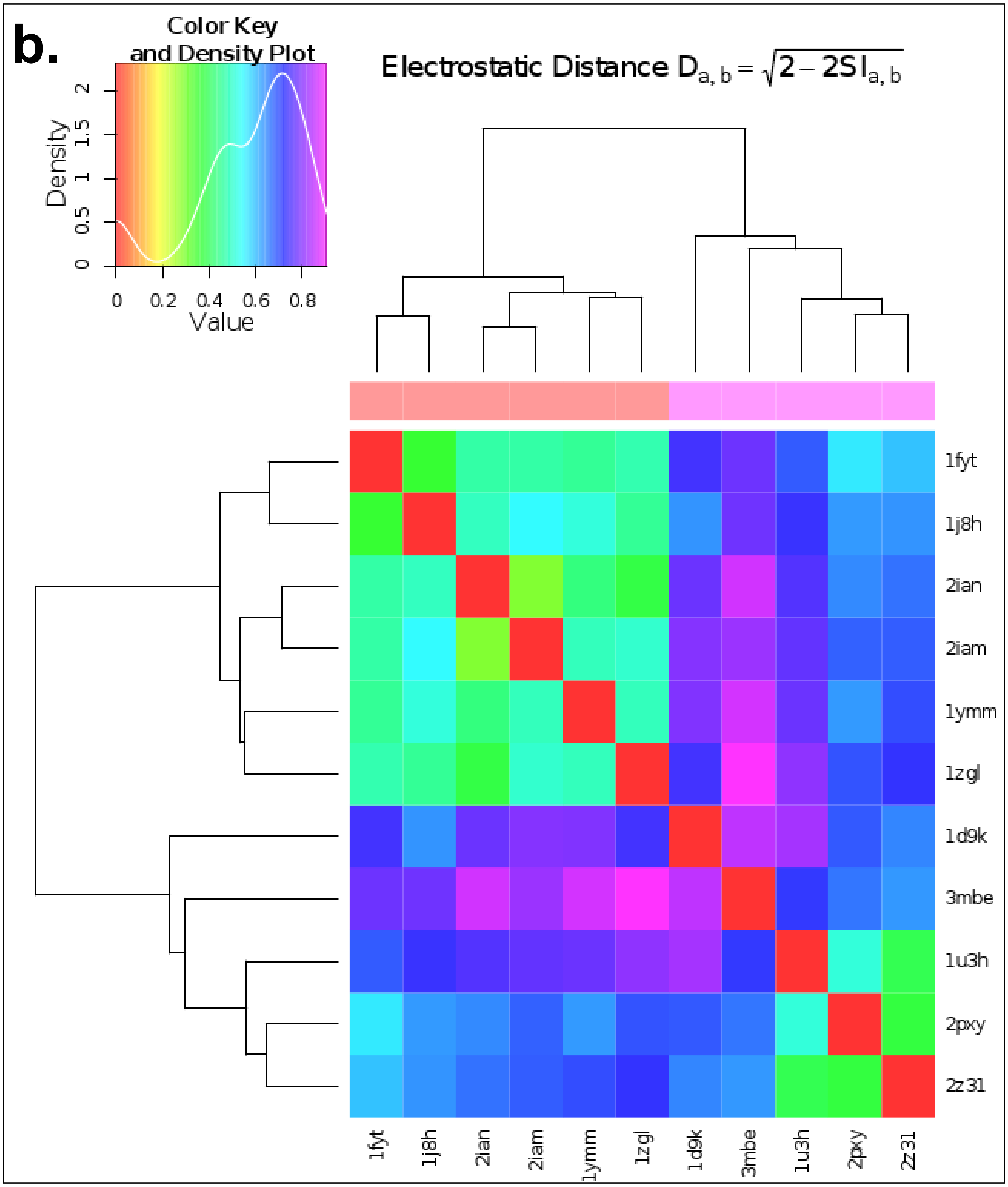

Figure S2. Heat maps for all pMHC interfaces based on the calculated MSEP values depicted as a colour coded matrix showing clustering amongst pMHC complexes in a reverse order as compared to the cluster dendograms in Supplementary Figure 1. a. pMHC-I complexes clustered into three. b. pMHC-II structures in two distinct clusters. Each pMHC interface is again denoted by its corresponding PDB code. Inset, are the legends showing the color key used to create heat matrices and the MSEP value ranges for pMHC interfaces. Also shown is the formula used to calculate electrostatic distances for clustering.
